# Beyond β-Lactams: Defining the Role of Eravacycline in Multidrug-Resistant and Metallo-β-Lactamase-Producing Infections

**DOI:** 10.3390/antibiotics15050503

**Published:** 2026-05-18

**Authors:** Jacob M. Keck

**Affiliations:** Department of Pharmacy, University of Arkansas for Medical Sciences (UAMS), Little Rock, AR 72205, USA; jmkeck@uams.edu

**Keywords:** eravacycline, New Delhi metallo-β-lactamase (NDM), multidrug-resistant (MDR) pathogens

## Abstract

The global spread of multidrug-resistant (MDR) Gram-negative pathogens has significantly narrowed therapeutic options for serious infections. MDR organisms frequently harbor multiple resistance mechanisms, such as β-lactamases and non-β-lactam determinants, which limit the activity of many β-lactam/β-lactamase inhibitor combinations and complicate the clinical utility of newer agents such as cefiderocol and aztreonam–avibactam. These challenges highlight the need for mechanistically distinct, non-β-lactam therapies capable of maintaining activity in MDR settings. Eravacycline is a fully synthetic fluorocycline antibiotic that inhibits bacterial protein synthesis through high-affinity binding to the 30S ribosomal subunit, a mechanism unaffected by β-lactamase-mediated resistance. Structural modifications at key positions confer stability against common tetracycline resistance mechanisms, including efflux pumps and ribosomal protection proteins. In vitro surveillance studies consistently demonstrate potent activity against a broad range of MDR Gram-negative pathogens, notably carbapenem-resistant Enterobacterales and isolates harboring metallo-β-lactamases. The clinical efficacy and safety of eravacycline have been established in pivotal Phase 3 trials for complicated intra-abdominal infections. Although highly resistant phenotypes were underrepresented in these trials, emerging real-world data describe off-label use in MDR Gram-negative infections, often as salvage or step-down therapy. These experiences suggest acceptable clinical outcomes and favorable tolerability in complex, high-risk patients. This review synthesizes mechanistic, microbiologic, pharmacologic, and clinical evidence supporting eravacycline’s potential role in the management of MDR Gram-negative infections.

## 1. Introduction

The global proliferation of multidrug-resistant (MDR) Gram-negative pathogens represents one of the most formidable challenges in contemporary infectious disease practices [1,2,3]. Among these, New Delhi metallo-β-lactamase (NDM)-producing organisms represent a particularly concerning subset due to their extensive resistance mechanisms and rapid global dissemination [4,5,6,7,8]. NDM enzymes confer high-level resistance to nearly all β-lactam antibiotics, and are frequently co-harbored with additional resistance determinants that further limit therapeutic options [9]. As a result, infections caused by NDM-producing Enterobacterales are associated with delayed effective therapy, increased reliance on toxic salvage regimens, increased financial burden, and poor clinical outcomes, particularly in critically ill patients [10,11].

Although several novel β-lactam/β-lactamase inhibitors (BLIs) combinations have expanded treatment options for MDR Gram-negative infections, their activity against metallo-β-lactamases (MBLs), particularly NDM-producing pathogens, remains intrinsically limited [12,13,14]. As a result, clinicians often rely on alternative agents such as polymyxins, aminoglycosides, tigecycline, cefiderocol, or aztreonam-containing combination regimens [15,16]. These therapies are frequently compromised by dose-limiting toxicity, inadequate penetration at sites of infection, complex administration requirements, particularly in the outpatient setting, and inconsistent clinical efficacy [15,17]. For instance, polymyxins are associated with clinically significant nephrotoxicity and neurotoxicity, while aminoglycosides are constrained by nephrotoxicity and limited tissue penetration in deep-seated infections [18,19]. Tigecycline has been associated with increased rates of gastrointestinal intolerance compared with eravacycline, as well as concerns regarding excess mortality in certain patient populations [20,21,22,23].

In this context, eravacycline, a fully synthetic fluorocycline antibiotic, has emerged as a potential therapeutic option for infections caused by MDR Gram-negative pathogens [24,25,26,27,28,29,30,31,32,33,34,35]. Eravacycline exerts its antibacterial activity through inhibition of bacterial protein synthesis via high-affinity binding to the 30S ribosomal subunit, a mechanism that is unaffected by β-lactamase production, including MBLs. Structural modifications relative to earlier tetracyclines confer enhanced stability against common resistance mechanisms such as efflux pumps and ribosomal protection proteins, resulting in potent in vitro activity against a broad range of MDR Gram-negative pathogens, notably carbapenem-resistant Enterobacterales (CRE) [36,37]. Additionally, eravacycline exhibits pharmacokinetic properties that support its clinical utility, characterized by extensive tissue distribution, high intra-abdominal penetration, and a favorable safety profile compared with alternative salvage agents [37,38,39]. However, despite these favorable pharmacologic characteristics, eravacycline is not currently U.S. Food and Drug Administration (FDA)-approved for indications beyond complicated intra-abdominal infections (cIAI). Clinical development programs evaluating eravacycline in other infection types did not demonstrate consistent noninferiority and did not lead to regulatory approval. Accordingly, discussion of its use outside of cIAI should be interpreted as exploratory and based on pharmacokinetic and microbiologic rationale rather than established, prospective clinical trial evidence [37,40,41,42].

This review examines the mechanistic rationale, in vitro activity, pharmacokinetic–pharmacodynamic characteristics, and clinical applicability of eravacycline for the treatment of infections caused by MDR Gram-negative pathogens, with a focus on NDM-producing organisms. By contextualizing eravacycline within the evolving therapeutic landscape of antimicrobial resistance, this review provides clinicians with a practical framework for its use in complex, high-risk infections.

This review was conducted as a narrative synthesis of the available literature. Relevant studies were identified through searches of PubMed and Google Scholar using combinations of keywords related to eravacycline, MDR Gram-negative pathogens, and MBL-producing organisms. Additional references were identified through manual review of bibliographies from selected articles. Study selection was guided by clinical relevance and the availability of microbiologic, pharmacokinetic–pharmacodynamic, and clinical outcome data.

## 2. The Expanding Challenge of Multidrug-Resistant Gram-Negative Infections: Metallo-β-Lactamases and Emerging Therapeutic Limitations

The global rise in MDR Gram-negative pathogens has fundamentally altered the management of serious infections, with NDM-producing organisms representing a particularly concerning subset [4,7,13,43]. Since its initial description, NDM has disseminated rapidly across both healthcare and community settings, driven by plasmid-mediated transmission and international patient movement. NDM-producing Enterobacterales are now increasingly encountered in severe infections and are associated with limited therapeutic options and adverse clinical outcomes [4,5,7,10,11,44,45,46,47,48,49,50]. Unlike other carbapenemases, NDM confers resistance to nearly all β-lactams, while frequently coexisting with additional resistance mechanisms that further restrict treatment options [6,7,8,12,13,15,47,48,50,51,52]. As a result, infections caused by NDM-producing organisms are frequently associated with delayed initiation of effective therapy and increased reliance on agents with less favorable safety profiles, both of which contribute to worse clinical outcomes [10,45,53].

A defining feature of MBL-producing organisms is their frequent co-expression of multiple β-lactamases from different Ambler Classes, resulting in complex and often unpredictable resistance phenotypes [7,12,46,54]. Class A β-lactamases include serine-based enzymes such as extended-spectrum β-lactamases and carbapenemases, which hydrolyze penicillins, cephalosporins, and, in some cases, carbapenems, and are variably inhibited by BLIs. Class C enzymes, primarily AmpC β-lactamases, confer resistance to many cephalosporins and are not reliably inhibited by most traditional BLIs. Class D β-lactamases, predominately OXA-type carbapenemases, exhibit variable hydrolytic activity against carbapenems and frequently coexist with other resistance mechanisms. In contrast, Class B β-lactamases (MBLs) are zinc-dependent enzymes that hydrolyze nearly all β-lactams except monobactams (aztreonam) and are not inhibited by any commercially available BLIs. This fundamental mechanistic distinction renders many β-lactam-based strategies ineffective when MBLs are present [12,13,52,54,55,56]. As a result, β-lactam susceptibility results may not reliably predict clinical response in the presence of MBLs and co-produced β-lactamases, particularly in severe or high-inoculum infections, thereby complicating therapeutic decision-making [12,57,58,59,60]. These challenges highlight the limitations of traditional β-lactam-based strategies and underscore the need for alternative therapeutic approaches.

Given these therapeutic challenges, rapid genotypic identification of resistance determinants is increasingly essential for optimal antimicrobial selection [12,61,62,63,64,65,66,67,68,69]. Phenotypic susceptibility testing alone may fail to capture the clinical implications of the presence of MBLs or multiple co-expressed β-lactamases. Molecular diagnostics capable of rapidly detecting MBLs and associated resistance genes allow clinicians to anticipate β-lactam failure, avoid ineffective therapies, and consider alternative non-β-lactam agents earlier in the course of infection [12,63,64,65,66,67,68,69]. In an era of escalating resistance and diminishing therapeutic margins, timely genotypic testing is a critical component of effective clinical management for infections caused by MDR Gram-negative pathogens, particularly those producing MBLs [12,67,68,69].

## 3. Contemporary β-Lactam-Based Therapeutic Strategies for NDM-Producing Pathogens

Despite the expanding availability of novel antimicrobial agents, the treatment of infections caused by NDM-producing organisms remains uniquely challenging [12,13,14]. β-lactam-based strategies continue to dominate clinical practice; however, their effectiveness in the setting of MBL production is limited by complex resistance mechanisms, ongoing bacterial adaptation, and practical constraints in real-world use [12,13,14,54]. Among currently available options, cefiderocol and aztreonam–avibactam (ATM-AVI) represent key β-lactam-based approaches for NDM-associated infections, each with distinct mechanistic advantages and important limitations [12,13,14,70,71].

### 3.1. Cefiderocol

Cefiderocol is a novel catechol-substituted siderophore cephalosporin specifically engineered to exploit bacterial iron acquisition pathways to enhance penetration into Gram-negative pathogens. By chelating extracellular ferric iron, cefiderocol forms a stable iron–drug complex that is actively transported across the outer membrane via TonB-dependent siderophore uptake systems such as CirA, Fiu, and related iron transport receptors. This “Trojan horse” mechanism enables cefiderocol to bypass traditional porin-mediated entry pathways and overcome common permeability-based resistance mechanisms, facilitating high periplasmic drug concentrations even in organisms with reduced outer membrane porin expression [72,73,74,75,76,77,78,79,80]. Structurally, cefiderocol combines this siderophore-mediated uptake with a cephalosporin core that demonstrates in vitro stability against hydrolysis by many β-lactamases across all Classes A–D. These properties initially positioned cefiderocol as a potential therapeutic option for infections caused by highly resistant Gram-negative pathogens; however, clinical outcomes have been variable in certain high-risk populations [72,73,76,77,78,79,80,81,82].

From a pharmacokinetic–pharmacodynamic standpoint, cefiderocol exhibits classic β-lactam time-dependent bactericidal activity, with antibacterial efficacy most closely linked to maintaining free drug concentrations above the minimum inhibitory concentration (MIC) for a sufficient proportion of the dosing interval (fT > MIC) [72,76,77,79]. Standard dosing regimens employ prolonged infusions to optimize pharmacodynamic target attainment, particularly in critically ill patients with altered volume of distribution or augmented renal clearance [72,76,77,79,80]. Cefiderocol exhibits favorable tissue penetration, achieving measurable and clinically relevant concentrations in epithelial lining fluid, urine, and intra-abdominal compartments, supporting its FDA-approved indications for hospital-acquired bacterial pneumonia, ventilator-associated bacterial pneumonia and complicated urinary tract infections [72,77,79,80]. Importantly, however, cefiderocol displays moderate protein binding and complex iron-dependent pharmacology, introducing variability in free drug exposure that may not be fully captured by conventional susceptibility testing methodologies [72,83,84].

Despite its mechanistic appeal, increasing reports of cefiderocol resistance among NDM-producing organisms have tempered early enthusiasm [85,86,87,88,89]. Resistance has been described both following clinical exposure and, notably, in cefiderocol-naïve isolates, suggesting potential intrinsic limitations related to the drug’s reliance on intact iron uptake systems [32,72,85,86,88,89,90,91]. Proposed resistance mechanisms include mutations or loss of siderophore receptor proteins, downregulation of iron transport pathways under iron-replete conditions, alterations in outer membrane permeability, and adaptive metabolic shifts that reduce cefiderocol uptake. Additionally, co-expression of other β-lactamases and target site modifications may synergistically impair activity despite nominal in vitro susceptibility [72,85,86,88,89]. These resistance pathways are particularly relevant in NDM-producing pathogens, which frequently harbor multiple resistance determinants and exhibit highly plastic regulatory responses to environmental stress [72,85,86,88,89]. Clinically, treatment failures have been reported despite cefiderocol MICs within the susceptible range, raising concerns regarding the reliability of standard in vitro susceptibility testing to predict in vivo efficacy in the context of MBL-mediated resistance [69,70,77,78]. These findings suggest that cefiderocol effectiveness may be reduced in complex resistance settings, raising concerns that reliance on cefiderocol monotherapy, especially in empiric therapy, may increase the risk of inadequate coverage and treatment failure.

### 3.2. Aztreonam–Avibactam (ATM-AVI)

ATM-AVI represents a mechanistically rational β-lactam-based strategy specifically designed to overcome the intrinsic limitations of β-lactamase inhibition in infections caused by NDM-producing organisms [92,93,94,95,96,97,98]. Aztreonam is uniquely stable to hydrolysis by MBLs due to its monobactam structure and lack of interaction with zinc-dependent catalytic sites. However, aztreonam is readily hydrolyzed by co-expressed serine β-lactamases, which, as previously mentioned, are frequently co-harbored on mobile genetic elements in NDM-producing Enterobacterales [13,14,54,97,99]. Avibactam is a BLI that provides potent inhibition of Class A and C β-lactamases and variable activity against certain Class D enzymes (OXA-48), thereby protecting aztreonam from enzymatic degradation and restoring antibacterial activity against otherwise resistant phenotypes [13,14,54,93,94,95].

From a pharmacodynamic perspective, ATM-AVI retains the time-dependent bactericidal activity characteristic of β-lactam antibiotics, with efficacy most closely associated with the proportion of the dosing interval during which free aztreonam concentrations exceed the MIC (fT > MIC). Although the recent FDA approval of fixed-dose ATM-AVI has addressed prior availability barriers, the regimen remains pharmacokinetically complex [94,100,101]. Optimal activity depends on sustained, temporally aligned avibactam exposure to continuously inhibit co-produced serine β-lactamases [96]. In critically ill patients, factors such as augmented renal clearance, dynamic changes in volume of distribution, and organ dysfunction can lead to unpredictable drug exposure, increasing the risk of transient avibactam underexposure and functional “unmasking” of aztreonam to β-lactamase-mediated hydrolysis [60,94,100,101,102,103].

Beyond pharmacologic considerations, ATM-AVI remains operationally burdensome in real-world clinical practice. Prolonged infusion requirements, frequent dosing intervals, and high cumulative β-lactam exposure contribute to increased line utilization, nursing workload, and potential administration errors [92]. From an antimicrobial stewardship standpoint, extended courses of ATM-AVI therapy raise concerns regarding collateral microbiome disruption and selective pressure for resistance [104,105,106,107,108,109,110]. Emerging resistance to ATM-AVI has already been described, mediated by mechanisms such as porin channel loss, penicillin-binding protein modifications, and stepwise increases in aztreonam MICs, highlighting potential limitations of reliance on layered β-lactam inhibition [105,106,107,109,110,111]. Moreover, while ATM-AVI is increasingly used for severe, life-threatening infections, its logistical burden and infusion complexity continue to limit feasibility in outpatient parenteral antimicrobial therapy settings, often prolonging hospitalization and delaying discharge [32,112]. Collectively, these pharmacodynamic, resistance, and practical constraints highlight the need for complementary non-β-lactam strategies that provide durable activity against NDM-producing pathogens without dependence on continuous β-lactamase inhibition.

### 3.3. Clinical Implications and Unmet Needs

Cefiderocol and ATM-AVI exemplify both the meaningful therapeutic advances and the persistent vulnerabilities that define the current management of infections caused by NDM-producing Gram-negative pathogens. Cefiderocol offers the theoretical advantage of a β-lactam-based monotherapy approach, supported by favorable pharmacokinetics and broad tissue penetration [73,74,76,78,79,81]. However, increasing reports of resistance, variability in clinical efficacy despite in vitro susceptibility, and reliance on intact iron transport pathways have raised concerns regarding its clinical durability, particularly in genetically complex NDM-producing isolates that harbor multiple resistance determinants [7,85,87,89,104]. These uncertainties complicate clinical decision-making and limit confidence in cefiderocol as a stand-alone option for prolonged therapy.

In contrast, ATM-AVI provides a mechanistically rational approach that directly addresses the poly-β-lactamase environment characteristic of NDM-producing Enterobacterales [92,93]. By combining aztreonam’s intrinsic stability to MBLs with avibactam-mediated inhibition of co-expressed serine β-lactamases, this strategy restores activity against otherwise extensively drug-resistant phenotypes [93,95,98]. Nevertheless, the clinical application of ATM-AVI remains constrained by pharmacokinetic and operational complexity, requiring optimized infusion strategies, high cumulative β-lactam exposure, and frequent dosing [93,95,100]. These challenges are further amplified in critically ill patients with dynamic renal function and in outpatient settings, where prolonged or continuous infusions may be impractical, thereby limiting treatment feasibility and prolonging hospitalization [32,102,112].

Collectively, the limitations of cefiderocol and ATM-AVI underscore a broader unmet need for therapeutic options that provide reliable activity against NDM-producing pathogens without dependence on β-lactamase inhibition or specialized uptake pathways. Non-β-lactam agents with predictable pharmacokinetic–pharmacodynamic profiles, favorable tolerability, and simplified administration may offer critical advantages in scenarios where prolonged therapy, step-down treatment, or outpatient management is required [32]. As resistance mechanisms continue to diversify and therapeutic margins narrow, the development and strategic deployment of such alternatives will be essential to improving outcomes in patients with NDM-mediated infections [13,113].

## 4. Mechanistic Rationale and Clinical Positioning of Eravacycline in Multidrug-Resistant Gram-Negative Infections

### 4.1. Mechanistic Rationale and Resistance Stability

Eravacycline is a fully synthetic fluorocycline antibiotic that inhibits bacterial protein synthesis through high-affinity binding to the 30S ribosomal subunit, a mechanism fundamentally distinct from β-lactam-based agents [36,37,39,114]. By targeting ribosomal function rather than cell wall synthesis, eravacycline is unaffected by β-lactamase activity, and retains activity against a broad range of MDR Gram-negative pathogens [24,25,29,36,115,116,117]. Because NDM mediates resistance through enzymatic degradation of β-lactam antibiotics, eravacycline’s antibacterial activity is preserved regardless of NDM expression level or the presence of additional carbapenemases. This mechanistic independence from β-lactam pathways positions eravacycline as a potential non-β-lactam therapeutic option in settings where β-lactam-based strategies are limited [44,115,117].

Structural modifications unique to eravacycline further distinguish it from earlier tetracyclines and from tigecycline. Substitutions at the C7 and C9 positions enhance ribosomal binding affinity while increasing stability against common tetracycline resistance mechanisms, notably efflux pumps (e.g., Tet(A), Tet(B)) and ribosomal protection proteins (e.g., Tet(M)) [22,36,37,39,115,116,117,118,119,120]. These modifications translate into consistently lower MICs against MDR Enterobacterales compared with tigecycline in multiple surveillance datasets [22,23,120,121,122,123]. This enhanced in vitro potency has been observed across diverse MDR phenotypes, such as carbapenem-resistant and MBL-producing isolates. However, the relationship between MIC values and clinical outcomes in infections caused by highly resistant organisms remains incompletely defined, particularly in NDM-producing organisms, which frequently co-harbor resistance determinants across multiple antibiotic classes [12,48,54,124,125,126,127,128,129].

Despite these uncertainties, a key advantage of eravacycline’s in vitro profile is its retained activity despite co-expressed resistance mechanisms, which are common among MDR-producing organisms [12,25,116,130,131,132,133]. These pathogens frequently harbor multiple resistance determinants, with β-lactamases, aminoglycoside-modifying enzymes, and fluoroquinolone resistance mutations commonly observed [12,15,48,130,131,134,135]. Additionally, eravacycline’s structural modifications allow it to evade tetracycline-specific efflux pumps (e.g., Tet(A), Tet(K)) and ribosomal protection proteins [36,37,39,132,136]. Collectively, these properties support preserved activity in isolates resistant to older tetracyclines and, in some cases, tigecycline [118,132,136].

### 4.2. In Vitro Activity and MIC Considerations

Eravacycline demonstrates in vitro activity against a broad range of MDR Gram-negative pathogens (see Section 4.4 for a detailed discussion of the spectrum of activity) [32,36,116,131,137,138]. Surveillance studies have shown that eravacycline retains activity against isolates harboring diverse resistance mechanisms, such as extended-spectrum β-lactamases, carbapenemases, and efflux-mediated resistance pathways [30,115,116,130,131,132,139,140]. Compared with tigecycline, eravacycline consistently exhibits lower MICs across multiple datasets, suggesting greater in vitro potency against resistant Enterobacterales [116,130,132,139].

To better contextualize these findings, MIC distributions from selected representative surveillance studies are summarized in Table 1 [116,131,137,141,142,143,144,145]. Across these representative datasets, eravacycline MIC_50_ and MIC_90_ values against Enterobacterales are generally low, with several studies demonstrating lower MIC values compared to tigecycline, particularly among subsets of carbapenem-resistant and MBL-producing isolates. These findings are consistent with additional in vitro studies cited throughout this review, which similarly demonstrate favorable activity of eravacycline relative to tigecycline across MDR Gram-negative pathogens [37,109,115,123,132,139,140,145,146]. However, variability in MIC distributions is observed depending on organism, resistance genotype, and study methodology, and underscores the importance of interpreting these data within a clinical context.

Despite favorable in vitro potency, the relationship between MIC values and clinical outcomes in infections caused by MDR Gram-negative pathogens remains incompletely defined. Available data suggest that microbiologic susceptibility does not uniformly translate into clinical success, particularly in infections characterized by high bacterial burden, limited source control, or altered pharmacokinetics in critically ill patients [129,147,148,149,150,151]. In MDR Gram-negative pathogens, where resistance mechanisms are layered and dynamic, MIC values may not fully capture the complexity of antimicrobial activity in vivo, particularly in patients with altered pharmacokinetics [148,149,152,153]. Accordingly, reliance on MIC data alone may be insufficient to guide optimal therapeutic selection in these clinical settings.

Interpretation of eravacycline susceptibility is further complicated by the limited availability of standardized breakpoints across organisms and infection sites. While regulatory agencies have established susceptibility criteria for select pathogens, these breakpoints may not fully account for variability in tissue penetration, site-specific pharmacodynamics, or the presence of complex resistance mechanisms [116,123,154]. In addition, differences in testing methodologies across surveillance studies may contribute to variability in reported MIC values, further complicating direct comparisons between datasets. These limitations highlight the need for cautious interpretation of in vitro susceptibility results, particularly when extrapolating to clinical decision-making in patients with MDR infections. Additionally, polymyxins and aminoglycosides, historically relied upon for the treatment of carbapenem-resistant infections, increasingly demonstrate limitations such as elevated MICs, heteroresistance, challenges with susceptibility testing (particularly for polymyxins), and unpredictable pharmacodynamics [136,155,156,157,158,159,160,161,162,163,164,165,166,167]. In contrast, eravacycline has demonstrated relatively stable MIC distributions across geographic regions and resistance phenotypes, with a more favorable safety profile that avoids the nephrotoxicity and neurotoxicity commonly associated with polymyxins and aminoglycosides [22,25,26,27,28,29,36,38,42,123,131,132,137,168]. These distinctions highlight potential advantages of eravacycline within the evolving treatment landscape of MDR Gram-negative infections.

### 4.3. Pharmacokinetics and Pharmacodynamics of Eravacycline

From a pharmacokinetic–pharmacodynamic standpoint, eravacycline exhibits properties well suited for the treatment of deep-seated infections caused by MDR pathogens [36,38,39,115]. The drug demonstrates a large volume of distribution (~4–5 L/kg), extensive tissue penetration, and predictable exposure in skin, soft tissue, and intra-abdominal compartments. Antibacterial efficacy is best correlated with the ratio of the area under the concentration–time curve to the minimum inhibitory concentration (AUC/MIC), and standard dosing (1 mg/kg IV every 12 h) achieves exposures consistent with established pharmacodynamic targets for susceptible organisms [116,169].

Eravacycline is primarily eliminated via nonrenal pathways and does not require dose adjustment in renal impairment, which may be advantageous in critically ill patients with fluctuating renal function. In contrast to concentration-dependent agents such as aminoglycosides or polymyxins, eravacycline does not rely on high peak concentrations for efficacy, allowing for effective therapy without the same risk of dose-limiting nephrotoxicity or neurotoxicity [27,29,38,39,123,138,170]. Variability in drug exposure may still occur in critically ill patients due to altered physiology, underscoring the importance of clinical context when applying pharmacokinetic–pharmacodynamic principles [171,172].

### 4.4. Spectrum of Activity

Eravacycline’s broad antimicrobial spectrum further supports its role in complex infections where polymicrobial involvement is common. It demonstrates potent activity against Gram-negative pathogens, such as carbapenem-resistant *Escherichia coli* and *Klebsiella pneumoniae* [115,116,117,120]. Coverage extends to Gram-positive pathogens, notably methicillin-resistant *Staphylococcus aureus*, penicillin-resistant *Streptococcus pneumoniae*, and vancomycin-resistant *Enterococcus faecium* [116]. Importantly for cIAI, eravacycline retains reliable activity against anaerobes, such as *Bacteroides fragilis* and *Clostridioides* species, enabling monotherapy in many cIAI scenarios [40,41,42,115,116,117,120,123,140,168]. However, activity against non-fermenting Gram-negative pathogens is variable; while coverage of *Pseudomonas aeruginosa* is generally limited, activity against *Acinetobacter baumannii* and *Stenotrophomonas maltophilia* has been reported but remains inconsistent and dependent on both isolate-specific resistance mechanisms and patient-specific clinical factors [28,34,36,37,132,141,171,173,174,175,176].

### 4.5. Bacteriostatic Activity and Clinical Implications

Eravacycline, like other tetracycline derivatives, is generally considered bacteriostatic against most Gram-negative pathogens [37,38,39,131,177]. This pharmacodynamic characteristic has raised theoretical concerns regarding its use in high-inoculum infections where bactericidal agents are traditionally preferred [178]. These concerns are rooted in the potential for reduced bacterial clearance in settings with high organism burden or limited host immune function, particularly in critically ill patients [179,180,181,182]. However, the clinical relevance of bacteriostatic versus bactericidal activity remains incompletely defined, and available evidence does not consistently demonstrate inferior outcomes with bacteriostatic agents in serious infections [27,38,183,184,185,186,187]. Clinical outcomes are likely influenced by multiple factors beyond bacteriostatic versus bactericidal activity, such as antimicrobial exposure, site of infection, source control, and host immune status [182,188]. Emerging real-world data and clinical experience suggest that eravacycline may still be effective in selected patients with MDR infections, although data specifically evaluating bloodstream and endovascular infections remain limited [178,183]. Accordingly, the use of eravacycline should be individualized based on infection source, organism susceptibility, and patient-specific factors [38,123]. In high-inoculum or endovascular infections, clinicians may consider combination therapy or alternative agents when appropriate, particularly in the setting of severe illness or inadequate source control [33,189]. Further studies are needed to better define the role of eravacycline in these higher-risk clinical scenarios and to clarify its optimal positioning within treatment algorithms for MDR infections.

### 4.6. Clinical Evidence and Limitations

#### 4.6.1. Randomized Clinical Trials

Eravacycline is FDA-approved for the treatment of complicated intra-abdominal infections (cIAI) based on two Phase 3 randomized controlled trials, IGNITE-1 and IGNITE-4 [40,41,184]. IGNITE-1 demonstrated noninferiority of eravacycline compared with ertapenem, while IGNITE-4 established noninferiority versus meropenem for clinical cure at test-of-cure in hospitalized adults with cIAI [40]. Across both trials, eravacycline exhibited a favorable safety profile, with low rates of treatment discontinuation and a lower incidence of severe gastrointestinal adverse effects compared with historical tigecycline experience. Although these studies were not designed to evaluate carbapenemase-specific outcomes, they provide foundational evidence supporting eravacycline’s efficacy in infections caused by Gram-negative and anaerobic pathogens. NDM-producing organisms were uncommon in these trials, and extrapolation to these resistant phenotypes should be interpreted with caution.

While randomized clinical trials supporting eravacycline approval did not specifically target infections caused by NDM-producing organisms, accumulating microbiologic and pharmacologic data suggest that eravacycline retains activity across diverse MDR clinical contexts, particularly when β-lactam-based therapies are limited or not feasible [24,25,26,27,28,29,30,31,32,33,34,131,137,190]. However, clinical data specific to NDM-producing pathogens remain limited, and much of the available evidence is derived from broader MDR and carbapenem-resistant organism populations. As such, extrapolation of clinical outcomes to NDM-specific infections should be interpreted cautiously [24,25,26,27,28,29,30,31,32,33,34,35,38,39,41,42,123,130,133,142,154,169,171,173,174,176,189,191,192,193,194,195,196,197]. Please see Table 2 for a summary of the IGNITE-1 and IGNITE-4 trials, as well as the pooled analysis from both studies.

#### 4.6.2. Real-World Experience in MDR and NDM-Producing Infections

Clinical evidence evaluating eravacycline for infections due to CRE, CRAB, MDR *Stenotrophomonas maltophilia*, and NDM-producing organisms remains largely observational but continues to expand. Retrospective cohorts and case series describe its use across a range of severe MDR infections, such as cIAI, skin and soft tissue infections, and ventilator-associated pneumonia, often in critically ill or immunocompromised patients [24,25,26,29,30,32,35,118,121,130,132,139,171,174,189,193,197,198]. Across these studies, clinical success rates are variable but generally favorable, though interpretation is limited by heterogeneity in study design, infection types, and outcome definitions. Eravacycline has been used both as monotherapy and in combination regimens, frequently in the setting of salvage therapy or prior treatment failure, with emerging data suggesting that outcomes may be influenced by factors such as early initiation and adequate source control [27,34,171,173]. Importantly, most available studies include mixed populations of carbapenem-resistant organisms, and relatively few specifically report outcomes in genotypically confirmed NDM-producing isolates, limiting direct extrapolation to NDM-specific infections [32,171].

More recent literature has focused on combination strategies and pharmacokinetic–pharmacodynamic optimization of eravacycline for MDR-producing Gram-negative pathogens. Preclinical and translational studies demonstrate additive or synergistic activity when eravacycline is combined with agents such as polymyxins, aminoglycosides, and aztreonam-based regimens targeting these pathogens [28,33,35,171,176,189,194,199,200]. Limited clinical data further support a potential role for combination therapy in severe infections, although these findings remain observational and hypothesis-generating [28,33,171,189,194]. Collectively, current evidence supports eravacycline as a potential adjunctive option in the management of MDR infections, but prospective studies are needed to better define its optimal role and comparative effectiveness. A summary of key clinical studies evaluating eravacycline in MDR infections is provided in Table 3.

#### 4.6.3. Limitations of Existing Clinical Data

Although real-world data provide encouraging results, particularly for carbapenem-resistant infections, most available evidence is retrospective, involves relatively small patient cohorts, and frequently lacks systematic carbapenemase genotyping (see Table 3). In addition, the common use of combination antimicrobial therapy complicates attribution of clinical outcomes to eravacycline alone, particularly in infections involving CRAB. Collectively, these limitations underscore the need for prospective studies and more granular microbiologic data to better define the role of eravacycline in infections caused by MDR-producing pathogens.

### 4.7. Role of Eravacycline in Therapy and Combination Considerations

The optimal role of eravacycline in the treatment of MDR Gram-negative infections continues to evolve and should be considered within the context of infection severity, pathogen susceptibility, and available therapeutic alternatives [123,201]. Based on current evidence, eravacycline is most appropriately positioned as a salvage or adjunctive agent, particularly in infections caused by organisms with limited treatment options or in patients unable to receive β-lactam-based therapies [24,25,26,27,28,29,30,31,32,33,34,35,48,171,173,174,176,189,193,194]. Its favorable tolerability profile, lack of nephrotoxicity, and predictable pharmacokinetics may offer advantages over agents such as polymyxins or aminoglycosides in select clinical scenarios, notably in patients with renal dysfunction [29,123]. While eravacycline has demonstrated efficacy in randomized trials for cIAI, its role as first-line therapy in infections caused by carbapenem-resistant or MBL-producing organisms remains less well defined, and β-lactam-based regimens are generally preferred when active and clinically appropriate [32]. However, eravacycline may represent a reasonable alternative in cases of intolerance, resistance, or logistical constraints, particularly when supported by in vitro susceptibility data [32].

**Table 3:** Review of pertinent eravacycline studies since 2020 (excluding meta-analysis).

Study (Year)	Design	Primary Pathogen Group	NDM Status	Infection Type(s)	Patient Population	Clinical Context	^#^ ERV USE	^^,&^ Key Clinical Outcomes
Van Hise et al. (2020) [24]	Retrospective, observational, cohort	Mixed MDR organisms (incl. KPC, *Acinetobacter*)	Not Reported	cIAI, PNA, DFI, SSTI	*n* = 50; high comorbidity burden (88% ≥ 2 comorbidities)	Real-world inpatient + OPAT; polymicrobial infections ~48%	* Monotherapy predominant	Clinical success 94%; mortality 0%; recurrence NR; AE 4%
Hobbs et al. (2022) [31]	Retrospective, multicenter, Observational cohort	Mixed MDR organisms (incl. CRE, CRAB, VRE)	Not reported	PNA, SSTI, cIAI, B&J, BSI	*n* = 66; high acuity; 42% ICU; ≥2 comorbidities ~59%	Real-world inpatient; polymicrobial infections common (majority)	Monotherapy predominant (62%)	Clinical success 86.4%; mortality NR; recurrence NR; AE 4.5%
Alosaimy et al. (2022) [173]	Multicenter, retrospective, cohort	*Acinetobacter* *baumannii* (69% CRAB)	Not reported	PNA (major), SSTI, cIAI, BSI, B&J	*n* = 46; high acuity; 41% ICU; high comorbidity burden	Real-world inpatient; polymicrobial infections common (majority)	Combination therapy predominant (84%)	Clinical success NR; mortality 23.9%; recurrence 21.7%; AE 2.2%
Buckley et al. (2023) [174]	Retrospective, single-center case series	MDR *Acinetobacter* *baumannii* (100% CRAB)	Not reported	PNA (major), SSTI, BSI	*n* = 10; mixed acuity; ~50% ICU; significant comorbidities	Real-world inpatient; polymicrobial infections common (majority)	Combination therapy predominant (100%)	Clinical success NR; mortality 0%; recurrence NR; AE NR
Kunz Coyne et al. (2024) [27]	Retrospective, multicenter, observational cohort (19 U.S. centers)	Mixed MDR organisms (incl. CRE, CRAB, VRE	Not reported	PNA, BSI, SSTI, cIAI, urinary, B&J	*n* = 416; high acuity; 42.5% ICU; median CCI 4.5	Real-world inpatient; severe infections; frequent source control procedures; polymicrobial infections common (~38%)	Mixed (50% combination)	Clinical success 75.7%; mortality 5.3%; recurrence 5.5%; AE 9.4%
Alexander et al. (2024) [34]	Retrospective, single-center, case–control study	MDR *Acinetobacter baumannii* (100% CRAB); 91% ERV concomitant CRE	Not reported	SSTI, PNA, BSI	*n* = 11 (ERV arm; 4:1 matched to CMS *n* = 44); burn patients; severe injury (median TBSA ~40%)	Real-world inpatient burn center; polymicrobial infections predominant	** Combination therapy predominant	Clinical success 64%; mortality NR; recurrence NR; AE NR
Jackson et al. (2024) [33]	Retrospective, single-center, case series	MDR *Acinetobacter* *baumannii* (100% CRAB)	Not reported	VAP	*n* = 24; critically ill; 100% mechanically ventilated; COVID-19 population	Real-world ICU; polymicrobial infections predominant (75%)	Combination therapy predominant (100%)	Clinical success 71%; mortality 25%; recurrence NR; AE 0%
Chen et al. (2025) [28]	Retrospective, single-center, observational cohort	MDR *Acinetobacter* *baumannii* (100% CRAB)	Not reported	PNA (VAP)	*n* = 24; lung transplant recipients; high acuity; ICU population	Real-world inpatient transplant center; polymicrobial infections present (not fully quantified)	Combination therapy predominant (87%)	Clinical success 62.5%; mortality 16.7%; recurrence NR; AE NR
Giuliano et al. (2025) [29]	Retrospective single-center case series	Mixed MDR organisms (incl. CRE (KPC); VIM-producing Enterobacterales reported; no CRAB)	Not reported	cIAI, SSTI, UTI, BSI	*n* = 13; high acuity; majority with malignancy/ comorbidities	Real-world inpatient; polymicrobial infections 46.2%	Combination therapy predominant (84%)	Clinical success 69.2%; mortality 38.5%; recurrence NR; AE 7.7%
Luo et al. (2025) [30]	Retrospective multicenter observational cohort	Mixed MDR organisms (incl. CRAB, CRKP)	Not reported	HAP/VAP	*n* = 113; high acuity; 45.1% mechanically ventilated; multiple comorbidities	Real-world inpatient respiratory cohort; monomicrobial infections predominant (94.6%)	Combination therapy predominant (64%)	Clinical success 87.6%; mortality 8.0%; recurrence NR; AE 1.8%
Al Musawa et al. (2025) [35]	Retrospective multicenter observational cohort	*Stenotrophomonas maltophilia*	Not reported	PNA, cIAI, B&J, BSI, UTI, SSTI	*n* = 41; high acuity; 41.5% ICU; multiple comorbidities	Real-world inpatient multicenter cohort; polymicrobial infections (53%)	Monotherapy predominant (90%)	Clinical success 73.2%; mortality 31.7%; recurrence 4.9%; AE 9.8%
Mimram et al. (2025) [171]	Retrospective case series	DTR *Acinetobacter* *baumannii* (NDM-1 ± OXA-23)	NDM present	VAP (100%) ± BSI	*n* = 3; critically ill ICU; ARDS; multiorgan failure; ECMO use	Real-world ICU salvage therapy; polymicrobial not reported	Combination therapy predominant (66%)	Clinical success 66.7%; mortality 66.7%; recurrence NR; AE 0%
Keck et al. (2025) [32]	Case report	NDM-producing *Enterobacter cloacae* (CRE)	NDM present	B&J	*n* = 1; chronic orthopedic infection; hardware involvement; polymicrobial (bacterial + fungal)	Real-world inpatient to outpatient step-down therapy; polymicrobial infection (*E. cloacae* + *Candida parapsilosis*)	Combination therapy (ERV + antifungal; step-down from ATM-AVI)	Clinical success 100%; mortality 0%; recurrence 0%; AE 0%
Kunz Coyne et al. (2025) [26]	Retrospective multicenter observational cohort	Mixed MDR organisms (incl. CRE, VRE)	Not reported	BSI, PNA, SSTI	*n* = 82; immunocompromised; high acuity (67% ICU)	Real-world inpatient immunocompromised cohort; polymicrobial not reported	Monotherapy predominant; combination in subset	Clinical success 56.1%; mortality 31.7%; recurrence lower with timely ERV; AE NR
Patino et al. (2025) [25]	Retrospective multicenter observational cohort	Mixed MDR organisms (incl. VRE, CRE, CRAB, *Stenotrophomonas maltophilia*)	Not reported	PNA, UTI, SSTI, BSI	*n* = 48; high acuity; 54% ICU; 41% vasopressors; 33% immunocompromised	Real-world inpatient cohort; monomicrobial predominant (94%)	Combination therapy predominant (77%)	Clinical success 60.4%; mortality 39.6%; recurrence included in composite outcome; AE 4.2%
Guo et al., (2025) [189]	Retrospective single-center cohort	MDR *Acinetobacter* *baumannii* (100% CRAB)	Not reported	PNA (VAP)	*n* = 33 (ERV group); critically ill ICU; high comorbidity burden; MODS 45.5%	Real-world ICU cohort; polymicrobial infections predominant (>50% co-infection)	Combination therapy predominant (100%)	Clinical success 72.7%; mortality 15.2%; recurrence NR; AE 6.1%
Trizzino et al. (2026) [176]	Case series	DTR *Acinetobacter* *baumannii* (CRAB)	Not reported	cIAI, BSI	*n* = 2; critically ill; septic shock; high comorbidity burden; ICU-level care	Real-world salvage therapy after failure of multiple regimens; polymicrobial infections (100%)	Combination predominant (100%)	Clinical success 50%; mortality 50%; recurrence 0% (case 2); AE 0%
Wang et al. (2026) [193]	Retrospective multicenter observational cohort	Mixed MDR organisms (incl. CRAB, CRE, *Stenotrophomonas maltophilia*, MRSA, VRE)	Not reported	PNA, BSI, cIAI, UTI	*n* = 796; hematology patients; highly immunocompromised; high-risk (80% recent chemo; neutropenia common)	Real-world inpatient hematology cohort; monomicrobial predominant (92%)	Split almost 50/50 monotherapy vs. combination	Clinical success 88.8%; mortality 10.6%; recurrence NR; AE 2.5%
Li et al. (2026) [194]	Retrospective multicenter observational cohort	CRAB, *K. pneumoniae* (incl. CRE)	Not reported	PNA, BSI, cIAI, UTI, CNS	*n* = 1796; high acuity; 57.4% ICU; high comorbidity burden	Real-world inpatient cohort; monomicrobial predominant (100%)	Monotherapy predominant (75%)	Clinical success 82.6%; mortality 12.1%; recurrence 4.34%; AE 2.28%
Van Helden et al. (2026) [198]	Retrospective multicenter observational cohort	Enterobacterales (incl. CRE)	Not reported	cIAI, SSTI, BSI, PNA, UTI, B&J	*n* = 155; high acuity; 61% ICU	Real-world inpatient cohort; polymicrobial predominant (77%)	Monotherapy predominant (74%)	Clinical success 84.5%; mortality 13.5%; recurrence 1.3%; AE 8.4%

Abbreviations: AE (adverse effects); ATM-AVI (aztreonam–avibactam); ARDS (acute respiratory distress syndrome); B&J (bone and joint); BSI (bloodstream infection); CCI (Charlson Comorbidity Index); cIAI (complicated intra-abdominal infection); CMS (colistimethate sodium); CNS (central nervous system); CRAB (carbapenem-resistant *Acinetobacter baumannii*); CRE (carbapenem-resistant Enterobacterales); DFI (diabetic foot infection); DTR (difficult-to-treat and resistant); ECMO (extracorporeal membrane oxygenation); ERV (eravacycline); HAP (hospital-acquired pneumonia); ICU (intensive care unit); KPC (*Klebsiella pneumoniae* carbapenemase); MDR (multidrug-resistant); MODS (multiple organ dysfunction syndrome); *n* (number of patients); NDM (New Delhi metallo-β-lactamase); NR (not recorded); OPAT (outpatient parenteral antimicrobial therapy); PNA (pneumonia); SSTI (skin and soft tissue infection); UTI (urinary tract infection); VAP (ventilator-associated pneumonia); VIM (Verona integron-encoded metallo-β-lactamase), VRE (vancomycin-resistant enterococci). * Monotherapy use described as predominant; exact proportion not reported. ** Combination use inferred based on high rate of polymicrobial infections; not explicitly reported. ^#^ Monotherapy use was derived when not explicitly reported based on the absence of combination therapy. ^^^ Mortality definitions varied across studies (e.g., 30-day, in-hospital, or unspecified) and are reported as defined in each study ^&^ Clinical success reported as defined in each study; when not explicitly reported, it was derived from reported clinical failure.

Combination therapy has emerged as an important consideration in the management of severe infections caused by MDR pathogens, particularly in high-acuity or high-inoculum infections [27,28,29,30,31,33,35,171,174,193]. In vitro and translational studies suggest that eravacycline may exhibit additive or synergistic activity when combined with agents such as polymyxins and aminoglycosides for infections associated with MDR Gram-negative pathogens [142,200,202,203]. Clinical reports further support the use of combination regimens in select patients, where improved microbiologic clearance and mitigation of resistance emergence may be desirable, although definitive comparative data remain limited [27,28,29,30,31,33,35,142,171,173,174,193]. Accordingly, the decision to use eravacycline as monotherapy or in combination should be individualized based on infection site, organism, and patient-specific factors, with combination therapy considered in settings of severe illness, high bacterial burden, or complex resistance phenotypes.

### 4.8. Safety, Tolerability, and Clinical Positioning Relative to Tigecycline

The safety and tolerability profile of eravacycline represents a meaningful advancement over earlier tetracycline derivatives, particularly tigecycline [22,121]. Notably, tigecycline carries a FDA box warning for increased all-cause mortality observed in meta-analyses of clinical trials, whereas eravacycline has not demonstrated a similar signal and does not carry this warning [204]. In addition, rates of nausea and vomiting are lower than those historically reported with tigecycline, likely related to differences in dosing strategy and formulation [22,121]. Eravacycline has not demonstrated clinically significant QT prolongation or nephrotoxicity in clinical trials [40,41,123,193,205]. Furthermore, its pharmacokinetic profile supports convenient dosing strategies, and dose adjustments are recommended in patients with severe hepatic impairment [193,206]. Compared with older non-β-lactam agents such as polymyxins and aminoglycosides, eravacycline offers a more favorable tolerability profile, particularly with respect to nephrotoxicity and neurotoxicity [121,193,207]. In contrast, relative to β-lactam-based therapies, eravacycline has a distinct adverse effect profile, with some studies suggesting improved tolerability, although findings are mixed and direct comparative data remain limited [40,41,121,207]. Available observational data further suggest that clinical outcomes with eravacycline are generally comparable in selected MDR infections, supporting its consideration as an alternative in patients at risk for toxicity [24,25,26,27,28,29,30,31,33,34,35,142,171,173,174,176,189,193,194].

From an antimicrobial stewardship perspective, eravacycline presents a potential non-β-lactam option within the Standardized Antimicrobial Administration Ratio (SAAR) framework, particularly as a strategy to reduce reliance on broad-spectrum β-lactam agents [12,124]. As a non-β-lactam agent with activity against MDR Gram-negative pathogens, its targeted use may help reduce exposure to carbapenems, polymyxins, and combination β-lactam strategies that contribute to resistance selection pressure [12,36,37,39,40,41,42,118,123,168,190,208]. When deployed judiciously, guided by microbiologic data, eravacycline may serve as a stewardship-aligned alternative that preserves β-lactam agents while maintaining clinical efficacy in high-risk infections [12,32,37,40,41,118,124]. This approach may also reduce adverse effects associated with broad-spectrum β-lactam agents, specifically *Clostridioides difficile* infection [27,31,36,40,41].

Although eravacycline and tigecycline share a common tetracycline backbone, they occupy distinct roles in clinical practice [12,22,23,37,123,190,201,209]. Both agents retain in vitro activity against *Stenotrophomonas maltophilia*, although tigecycline is more commonly utilized in clinical settings [12,36,122,197]. For Acinetobacter spp., eravacycline often demonstrates enhanced in vitro potency compared to tigecycline; however, clinical data supporting its routine use for this indication remain limited and somewhat controversial [12,28,36,141,175,189,197]. Conversely, eravacycline exhibits improved potency against Enterobacterales, more favorable tolerability, and lower MIC distributions against many MDR Gram-negative pathogens [120,121,132,137,141]. These differences underscore the importance of selecting fluorocyclines based on pathogen-specific and infection-specific considerations, rather than viewing them as interchangeable agents. A comprehensive comparison of tigecycline and eravacycline, including key pharmacologic and clinical differences, is summarized in Table 4.

## 5. Conclusions

The global expansion of MDR pathogens continues to erode the utility of traditional β-lactam-based therapies and challenge existing antimicrobial development paradigms. Through its β-lactamase-independent mechanism of action, structural resilience to common resistance mechanisms, and favorable pharmacokinetic–pharmacodynamic properties, eravacycline represents a rational therapeutic option within this evolving landscape. Its robust in vitro activity against MDR Enterobacterales, demonstrated efficacy in cIAI, and emerging real-world experience collectively support its consideration in carefully selected clinical scenarios.

While eravacycline is not a panacea for MDR Gram-negative pathogens, it highlights the importance of incorporating non-β-lactam strategies into the broader management of these infections, particularly when resistance, toxicity, or logistical barriers limit conventional options. However, its role in the treatment of MDR pathogens remains incompletely defined, as current evidence is largely derived from observational studies and heterogeneous patient populations, including mixed carbapenem-resistant and MBL-producing cohorts. Accordingly, its use should be guided by susceptibility data, infection-specific considerations, and antimicrobial stewardship principles.

Continued investigation will be essential to better define the optimal clinical role of eravacycline and support its evidence-based integration into treatment strategies for MDR infections. Failure to appropriately incorporate agents such as eravacycline risks further narrowing an already limited therapeutic arsenal against these pathogens.

## Figures and Tables

**Table 1:** In vitro activity of eravacycline against MDR Enterobacterales.

Study	Organism	Resistance	Eravacycline MIC_50_ (mg/L)	Eravacycline MIC_90_ (mg/L)	Tigecycline MIC_50_ (mg/L)	Tigecycline MIC_90_ (mg/L)
Morrissey et al. 2020 [116]	Enterobacterales (10,531 isolates)	MDR (2051 isolates)	0.25	0.5	0.5	1
Hawser et al. 2023 [137]	Enterobacterales (12,436 isolates)	MDR (2931 isolates)	0.25–0.5	0.5–1	0.5	1
Additional Studies						
Study	Isolates	Organisms	Genotypes	Key Findings		
Brauncajs et al. (2023) [131]	102	MDR Enterobacterales	KPC, MBLs, OXA-48	ERV MIC_90_ KPC—1 ERV MIC_90_ MBLs—32 ERV MIC_90_ OXa-48—3		
Liao et al. (2024) [143]	594	GNB, MRSA, VRE, *A. baumannii*	Unknown	ERV MIC_90_ *E. coli*—0.5 ERV MIC_90_ *K. pneumoniae*—2 ERV MIC_90_ *A. baumannii*—2		
Bianco et al. (2025) [144]	264	CRKP	KPC	ERV MIC_90_ KPC—0.5 Tige MIC_90_ KPC—1		
Kinet-Poleur et al. (2025) [145]	222	MDR Enterobacterales	KPC, MBLs, OXA-48	ERV MIC_90_ [mg/L] Enterobacterales—1		
Chen et al. (2026) [141]	223	CRKP and CRAB	KPC, MBLs, OXA-48, OXA-23	ERV MIC_90_ CRKP—1 Tige MIC_90_ CRKP—4 ERV MIC_90_ CRAB—0.25 Tige MIC_90_ CRAB—1		
Zhao et al. (2026) [142]	42	CRKP	NDM, KPC-2	ERV MIC_90_ CRKP—1		

Abbreviations: *A. baumannii* (*Acinetobacter baumannii*); CRAB (carbapenem-resistant *Acinetobacter baumannii*); CRKP (carbapenem-resistant *Klebsiella pneumoniae*); *E. coli* (*Escherichia coli*); ERV (eravacycline); GNB (Gram-negative bacilli); *K. pneumoniae* (*Klebsiella pneumoniae*); KPC (*Klebsiella pneumoniae* carbapenemase); L (liter); MBLs (metallo-β-lactamases); MDR (multidrug-resistant); mg (milligrams); MIC (minimal inhibitory concentration); MRSA (methicillin-resistant *Staphylococcus aureus*); NDM (New Delhi metallo-β-lactamase); OXA (oxacillinase); Tige (tigecycline), VRE (vancomycin-resistant enterococci).

**Table 2:** Summary of major eravacycline randomized trial data for complicated intra-abdominal infections.

Trial	Study Design	Patient Population	Comparator	Primary Endpoint	Key Efficacy Results
IGNITE-1	Phase 3, randomized, double-blind, multicenter	Adults with cIAI	Ertapenem	Clinical cure at TOC in micro-ITT population; NI margin 10%	Clinical cure: 86.8% (eravacycline) vs. 87.6% (ertapenem); −0.80% (95% CI, −7.1% to 5.5%)
IGNITE-4	Phase 3, randomized, double-blind, multicenter	Adults with cIAI	Meropenem	Clinical cure at TOC in micro-ITT population; NI margin 12.5%	Clinical cure: 90.8% (eravacycline) vs. 91.2% (meropenem); −0.5% (95% CI, −6.3% to 5.3%)
Phase 3 Analysis	Pooled analysis of IGNITE trials	Adults with cIAI	SOC β-lactams	Clinical cure & microbiologic eradication	Consistent noninferiority across subgroups

Abbreviations: CI (confidence interval); cIAI (complicated intra-abdominal infection); IGNITE (investigating Gram-negative infections treated with eravacycline); micro-ITT (microbiological intent-to-treat); NI (noninferiority); SOC (standard of care); TOC (test of cure); β-lactam (beta-lactam).

**Table 4:** Eravacycline vs. tigecycline.

Feature	Eravacycline	Tigecycline
Drug class	Fully synthetic fluorocycline	Glycylcycline
FDA-approved indication	cIAI	cIAI, ABSSSI
Activity vs. NDM producers	Preserved	Preserved but higher MICs
In vitro Gram-negative and Gram-positive coverage	ESBL-producing Enterobacterales, CRE, VRE, *S. aureus* (including MRSA), anaerobes, * *Acinetobacter* (CRAB), * *Stenotrophomonas*	ESBL-producing Enterobacterales, CRE, VRE, *S. aureus* (including MRSA), anaerobes, *Acinetobacter* (CRAB), *Stenotrophomonas*
PK driver	AUC/MIC	AUC/MIC
Renal adjustment	None	None
Penetration	CNS, lungs, IAI, bone	CNS, lungs, IAI, bone
Adverse effects	Lower rates of N/V	Higher rates N/V
FDA BBW [204]	None	Increased all-cause mortality observed in meta-analyses of clinical trials
** Common dosing	1 mg/kg q12h—inpatient 1.5 mg/kg OPAT	100 mg LD then 50 mg q12h (can do 200 mg LD followed by 100 BID for severe infections)
Formulation	IV only	IV only
FDA breakpoints Enterobacterales	(S): ≤0.5 mcg/mL (R): ≥1 mcg/mL	(S): ≤2 mcg/mL (R): ≥8 mcg/mL
Potential clinical niche (including off-label usage)	MDR GN ABSSSI, cIAI, and B&J (including NDM-associated infections)	MDR GN ABSSSI, cIAI, and B&J (including NDM-associated infections) CRAB-related infections, MDR *Stenotrophomonas*-related infections

Abbreviations: ABSSSI (acute bacterial skin and skin structure infections); AUC (area under the curve); BID (twice a day); B&J (bone and joint), BBW (black box warning); cIAI (complicated intra-abdominal infections); CNS (central nervous system); CRAB (carbapenem-resistant *Acinetobacter baumannii*); CRE (carbapenem-resistant Enterobacterales); ESBL (extended-spectrum β-lactamases); FDA (U.S. Food and Drug Administration); GN (Gram-negative); IAI (intra-abdominal); h (hours); IV (intravenously); LD (loading dose); mcg (micrograms); MDR (multidrug-resistant); MIC (minimum inhibitory concentration); MRSA (methicillin-resistant *Staphylococcus aureus*); mL (milliliter); N/V (nausea and vomiting); NDM (New Delhi metallo-β-lactamase); OPAT (outpatient antibiotic therapy); q (every); VRE (vancomycin-resistant enterococci). * Not recommended for use in these pathogens per the IDSA 2024 Guidance on the Treatment of Antimicrobial-Resistant Gram-Negative Infections [12]. ** Eravacycline dose must be adjusted for Child–Pugh C.

## Data Availability

No new data were created or analyzed in this study. Data sharing is not applicable to this article.
